# Salivary testosterone in male and female youth with and without autism spectrum disorder: considerations of development, sex, and diagnosis

**DOI:** 10.1186/s13229-022-00515-4

**Published:** 2022-09-19

**Authors:** Rachael A. Muscatello, Emma Rafatjoo, Karan K. Mirpuri, Ahra Kim, Simon Vandekar, Blythe A. Corbett

**Affiliations:** 1 Department of Psychiatry and Behavioral Sciences, Vanderbilt University Medical Center, 1500 21st Avenue South, Nashville, TN 37212 USA; 2 Vanderbilt University, Nashville, TN USA; 3 Department of Biostatistics, Vanderbilt University Medical Center, Nashville, TN USA; 4 Vanderbilt Kennedy Center, Vanderbilt University Medical Center, Nashville, TN USA; 5 Department of Psychology, Vanderbilt University, Nashville, TN USA

**Keywords:** Autism, Testosterone, Pubertal development, Adolescence, Androgen

## Abstract

**Background:**

Puberty is characterized by significant physical, hormonal, and psychological changes, which may be especially challenging for individuals with autism spectrum disorder (ASD). Although the etiology of ASD remains uncertain, studies suggest imbalances in hormones, such as testosterone, may modulate the autism phenotype. While differences in fetal and postnatal testosterone have been reported, there is limited literature regarding testosterone variations during adolescence in ASD. We investigated morning salivary testosterone levels in youth with ASD and typical development (TD) to explore hypothesized differences, expecting elevated hormonal levels in ASD compared to TD.

**Methods:**

Youth with ASD (*n* = 140) and TD (*n* = 104), ages 10 to 13 years, were enrolled as part of a longitudinal study on pubertal development. Pubertal stage was determined by gold standard physical examination, and salivary testosterone was collected in the morning immediately upon waking and 30 min after waking and averaged across 3 days. Diagnostic (ASD/TD) and sex (male/female) differences, as well as interactions with age and puberty, were examined using robust linear mixed effect models.

**Results:**

Youth with ASD showed significantly elevated testosterone concentrations compared to same-age TD peers. After the inclusion of natural cubic splines to account for nonlinearity in age, a significant age-by-sex interaction emerged with distinct developmental slopes for males and females. At younger ages, females had higher testosterone, until about 11.5 years of age, when levels began to plateau, while male testosterone concentrations continued to rapidly increase and surpass females. As expected, more advanced pubertal development was associated with elevated testosterone. In contrast, no significant effect of parent-reported social communication symptoms was observed.

**Limitations:**

Limitations include an unequal sex distribution, non-representative sample (e.g., cognition and race/ethnicity), and inability to examine afternoon/evening testosterone due to detection limits.

**Conclusions:**

Testosterone may play a unique role in the presentation of ASD, especially during periods of dynamic hormonal changes including puberty. Inherent developmental (age, puberty) and sex-based (male, female) factors play a more prominent role in changes in testosterone levels during adolescence. Even so, future research is warranted to determine the differential expression and impact of exposure to excess testosterone during the pubertal transition for youth with ASD.

**Supplementary Information:**

The online version contains supplementary material available at 10.1186/s13229-022-00515-4.

## Background

Autism spectrum disorder (ASD) is a neurodevelopmental condition characterized by impairments in social communication and interaction, as well as restricted and repetitive behaviors [1]. As such, many individuals with ASD have difficulty tolerating and adapting to changes. Adolescence, including the pubertal development period, is marked by tremendous physiological, psychological, and social change [2]. For individuals with ASD, the uncertainty of pubertal development may be especially challenging, enhancing vulnerability during this dynamic transition. Furthermore, atypical physiological arousal and social stress during adolescence likely contribute to poor emotional health (e.g., [3, 4]), emphasizing the need to better understand this critical period, especially for vulnerable individuals such as youth with ASD.

### Adolescence and the hypothalamic–pituitary–gonadal (HPG) axis

Adolescence marks the developmental transition of juvenile social and cognitive processes to their adult versions. While often used interchangeably in describing adolescence, puberty uniquely refers to biological maturation, particularly that of sexual systems, and the physiological effects of resultant changes to the endocrine system, including activation of the HPG axis [5]. The HPG axis is a primary driver in pubertal development, critical to maintaining homeostasis, facilitating sexual maturation (e.g., [6, 7]), and secreting gonadal hormones such as testosterone or estradiol.

The HPG axis undergoes three particularly important periods of activation. First, gonadotropin-releasing hormone (GnRH) may be detected in the fetal hypothalamus as early as 15 weeks of gestation and modulates the downstream secretion of gonadotropins (follicle-stimulating hormone [FSH] and luteinizing hormone [LH]) from the anterior pituitary and sex hormones (testosterone and estradiol) from the gonads [8]. However, HPG activity decreases in later stages of pregnancy and resurges again postnatally in the first few months of life within a ‘minipuberty’ (e.g., [9–11]). During this period, a sexual dimorphism occurs, with higher LH levels in biologically male newborns and higher FSH levels in biologically female newborns (e.g., [12, 13]). Following postnatal activation, the HPG axis undergoes a period of functional quiescence that persists throughout childhood until the third phase of activation, namely, pubertal onset, in which LH, FSH, testosterone, and estradiol levels rise and facilitate complete sexual maturation [14].

Hormones produced by the HPG axis have an extensive network of receptors in the brain and in peripheral tissues. The levels of these hormones are tightly regulated by a complex feedback loop, which serves to maintain normal concentrations of hormones in the brain and periphery. In early pre- and perinatal development, these sex hormones contribute to sex-specific neural circuitry organization, while hormonal changes at puberty activate sex-specific behavior arising from neural differences (see organizational–activational hypothesis; [15]). Indeed, HPG axis activation has been shown to significantly impact neuronal growth, development, and modulation [7]. For example, the sex steroids (e.g., estradiol, testosterone, etc.) have been shown to modulate neurite growth and migration, as well as dendritic spine density, thus contributing to the development of sexually dimorphic brain regions (e.g., [16, 17]). Furthermore, in animal studies, the timing of testosterone secretion during adolescence is directly related to the expression of sex-specific social behaviors (e.g., mating) [18]. The timing of HPG activation throughout critical developmental periods may therefore have important implications for neurodevelopment and psychological functioning.

### The HPG axis and autism

Considering the role of sex steroids and neuroendocrine processes in influencing brain and behavior development, a focus of previous research has been on the early impact of androgen hormones (e.g., testosterone) in autism. Especially relevant to this exploration of testosterone in ASD is the diagnostic bias within the neurodevelopmental condition, with a recently estimated 3:1 ratio of males to females [19]. Furthermore, the Extreme Male Brain theory (EMB), an extension of the empathizing–systemizing (E–S) theory [20], posits that ASD can be characterized as an extreme exhibition of the male brain, in which individuals with ASD will tend to display lower scores on behavioral measures related to empathizing and average or above-average performance on measures related to systemizing [20, 21]. With the overlap of autism symptom presentation and psychological/behavioral characteristics often associated with the male brain, the role of hormonal factors in driving early development and presentation of symptoms associated with autism continues to garner interest within the field.

Investigations have explored variations in testosterone levels between individuals with ASD and TD**,** largely focusing on prenatal and organizational effects, leading to the development of theories surrounding the potential association between ASD and such hormones. The previously termed fetal androgen theory (now prenatal steroid theory; [22, 23] implicates prenatal effects of androgens on developing autism [24, 25], theorizing observed differences in trait presentation result from heightened exposure to androgens, such as testosterone, during fetal development [26]. Indeed, evidence suggests testosterone plays a role in human social and emotional behavior, with prenatal exposure serving as an important indicator of brain organization and development [24, 25]. For example, high fetal testosterone has been linked to poor eye contact [27], poor social relationships [28], and less empathy [29]. Furthermore, researchers analyzed fetal testosterone levels in amniotic fluid samples and compared them to the presence of ASD-related traits in TD toddlers between 18 and 24 months using the Quantitative Checklist for Autism in Toddlers (Q-CHAT), finding a significant positive relationship between fetal testosterone levels and Q-CHAT scores [30]. In children aged 6–10 years, autistic traits as reported by the parent were positively correlated with previously measured fetal testosterone [31]. However, a large longitudinal study examining the umbilical cord blood of 707 children found no correlation between testosterone concentrations and the number of autistic traits [32].

In contrast, studies examining differences in postnatal (activational) androgen effects in individuals with ASD compared to TD controls have produced more variable results. In favor of an elevated testosterone hypothesis, a sample of prepubertal youth (ages 3–4 and 7–9 years) demonstrated elevated levels of adrenal hormones dehydroepiandrosterone (DHEA) and androsterone in males and females with ASD relative to age-matched TD controls [33]. Furthermore, associations have been reported between plasma testosterone and problem behaviors in prepubertal males (3–15 years) with ASD [34–36]. Significantly elevated serum and bioactive testosterone levels have been found for adult females with ASD (after correcting for Body Mass Index and excluding participants on oral contraceptives), although the study found no difference between ASD and TD adult males [37]. Another study observed increased serum androstenedione, a precursor molecule of testosterone, in adults with ASD across both biological sexes [38]. In contrast, a small study of pre- and post-pubertal youth did not find evidence of testosterone or DHEA differences in ASD relative to TD [39], while a study of ASD males, ages 12–18, found lower levels of basal testosterone upon waking, and generally throughout the course of the diurnal rhythm [40]. Variability in findings on testosterone concentrations in ASD has led researchers to hypothesize that significant developmental effects contribute to observations of testosterone differences in ASD, where puberty may serve as a period of normalization for the observed elevated levels of prepubertal testosterone associated with ASD-related traits, especially for males [41].

To date, research has largely focused on the role of testosterone during the fetal development period (organizational) and future presentation of autism symptoms; therefore, less is known regarding any link between androgen levels and ASD symptoms and behavioral phenotypes later in development (activational effects), particularly during puberty and adolescence. The current study sought to examine morning salivary testosterone levels in a large, well-characterized sample of early adolescents to observe potential diagnostic (ASD vs. TD) and sex (male vs. female) differences at pre- and peri-pubertal stages. Furthermore, hormonal concentrations were examined in association with development (age, pubertal status) and social functioning to explore the extent to which altered HPG axis functioning may be related to symptoms of ASD in the early adolescent period. It was hypothesized that youth with ASD would exhibit higher morning testosterone levels compared to the TD group and both ASD and TD male participants would exhibit higher morning testosterone levels relative to female participants. Additionally, developmental effects were predicted such that increase in age and pubertal development would be associated with higher levels of testosterone. Moreover, it was predicted that results would reveal a positive association between elevated testosterone and more severe social symptoms.

## Methods

### Participants

Data were collected as part of a large, longitudinal study on pubertal development and stress [42]. The current study includes data from Year 1 enrollment when the children were between 10 and 13 years of age. In other words, the overarching aim of the larger study was to examine pubertal development (i.e., timing, tempo, and course) and stress (e.g., cortisol, heart rate) in youth with ASD compared to TD peers of comparable age. During Year 1, a similar number of participants in each group (ASD, TD) were recruited; however, a strict case–control matching was not employed. Of the total sample of 244 youth, 140 had ASD (median age = 11.2, IQR = 10.5, 12.2) and 104 with TD (median age = 11.6, IQR = 10.6, 12.6). The ASD group included 36 (25.7%) females and the TD group included 46 (44.2%) females. Descriptive statistics for the sample are provided in Table 1.

**Table 1:** Demographic statistics

	*N*	TD		ASD		Test statistic	*p* value
		(*N* = 104)		(*N* = 140)			
		Md	IQR	Md	IQR		
Age	244	11.6	(10.6, 12.6)	11.2	(10.5, 12.2)	*F*_1,242_ = 2.71	0.101^3^
GB stage	238	2	(1, 3)	2	(1, 3)	*F*_1,236_ = 0.04	0.835^3^
PH stage	238	2	(1, 3)	1	(1, 3)	*F*_1,236_ = 0.12	0.733^3^
SRS total T Score	240	46.0	(43.0, 51.5)	76.0	(69.0, 82.0)	*F*_1,238_ = 544.4	< 0.001^3^
BMI percentile	239	53	(30, 88)	69	(39, 96)	*F*_1,237_ = 6.12	0.014^3^
Waking T day 1	206	5.4	(2.1, 13.5)	7.8	(1.1, 27.6)	*F*_1,201_ = 1.70	0.193^3^
Post-waking T day 1	203	3.3	(0.3, 13.1)	5.5	(0.6, 17.0)	*F*_1,201_ = 1.84	0.176^3^
Waking T day 2	206	5.1	(1.7, 12.3)	6.2	(1.4, 28.5)	*F*_1,204_ = 1.45	0.230^3^
Post-waking T day 2	202	3.1	(0.6, 12.1)	6.1	(0.7, 15.8)	*F*_1,200_ = 2.81	0.095^3^
Waking T day 3	203	4.1	(1.4, 13.4)	8.1	(3.0, 29.9)	*F*_1,201_ = 5.01	0.026^3^
Post-waking T day 3	197	3.6	(1.0, 9.9)	5.4	(1.4, 16.7)	*F*_1,195_ = 1.91	0.168^3^

	*N*	Proportion		Proportion		Test statistic	
Sex: female	244	0.442 46/104		0.257 36/140		*Χ*^2^ = 9.17	0.002^2^
Race	244					*Χ*^2^ = 12.06	0.007^2^
White		0.856 (89/104)		0.814 (114/140)			
Black		0.019 (2/104)		0.121 (17/140)			
Asian/Pacific Islander		0.000 (0/104)		0.007 (1/140)			
Multiracial		0.125 (13/104)		0.057 (8/140)			

*N* is the number of non-missing values. ^1^Kruskal–Wallis, ^2^Pearson, ^3^Wilcoxon. TD, typical development; ASD, autism spectrum disorder; Md, median; IQR, interquartile range; GB, genital/breast; PH, pubic hair; SRS, social responsiveness scale; BMI, body mass index; T, testosterone (pg/ml)

Autism diagnosis was based on the Diagnostic and Statistical Manual-5 [1] and confirmed by: (1) a previous diagnosis by a psychologist, psychiatrist, or behavioral pediatrician with autism expertise; (2) current clinical judgment, and (3) corroborated by the Autism Diagnostic Observation Schedule (ADOS-2; [43]) a semi-structured interview-based instrument administered by research-reliable personnel. Youth with ASD with a known genetic etiology (e.g., Fragile X syndrome) were excluded from enrollment. While genetic testing was not employed, it is suspected that the majority of the sample was idiopathic. The majority of the sample (*N* = 93) had a previous diagnosis, yet the age of diagnosis was not tracked. Additional inclusion criteria included a required intelligence quotient score (IQ) ≥ 70 for both ASD and TD groups, where IQ was estimated using the Wechsler Abbreviated Scale of Intelligence, Second Edition (WASI-II, [44]). Parents of youth in both groups completed the Social Communication Questionnaire (SCQ; [45]) to screen for symptoms of ASD; TD youth had to receive a score < 10 in order to be included in the study.

As part of the longitudinal study criteria, participants were not included if they reported any current use of medications known to alter the hypothalamic–pituitary–adrenal (HPA) axis (e.g., corticosteroids; see [46]) or HPG axis (e.g., growth hormone). Youth with any medical condition known to impact pubertal development (e.g., Cushing’s Disease) were excluded, as were participants taking oral contraceptives, growth hormones, or nicotine all known to influence the HPA and/or HPG axis [47, 48]. Of the total sample, 65.2% of youth in the ASD group were taking at least one medication, while 17.5% of TD participants reported taking daily medication. Medication use included stimulants, melatonin, selective-serotonin reuptake inhibitors, antihistamines, and central alpha-agonists.

The current study was carried out in accordance with the Code of Ethics of the World Medical Association (Declaration of Helsinki) and approved by the Vanderbilt Institutional Review Board (IRB). Informed written consent/assent was obtained from all care providers and study participants, respectively, prior to inclusion in the study. Participation required two research visits to the University. At visit 1, diagnostic and cognitive measures were administered to confirm study eligibility. During this visit, participants and their families were given instructions on collecting salivary samples at home (details below) and allowed the opportunity to practice to ensure understanding of the technique prior to leaving the lab.

### Pubertal and psychosocial measures

Recent research with the sample comparing physical examination to parent- and self-report demonstrated that physical examination is the optimal approach for more precise pubertal measurement [49]. Therefore, physical examination scores were used in the current study.

#### Physical examination (PE)

Physical examination procedures were followed according to the protocol previously reported by Corbett et al. [49]. In short, the PE was completed by a trained, licensed study physician to reliably identify pubertal development and assign Tanner stage [50, 51]. The examination ascertained two measures with 5 stages for genitals (G1–G5 for boys), breasts (B1–B5 for girls; GB stage), and pubic hair (P1–P5 for both genders; PH stage). The examination consisted of visual inspection and categorization of pubertal and genital maturation. To be consistent with the original Tanner staging and to maximize participation, palpation of breasts or measurement of testes was not conducted. As part of the larger study, participants provided self-reported perceived physical status, while parents reported on child’s pubertal development. Physicians were blinded to self- and parent reports.

#### Social responsiveness scale (SRS-2) [52]

The SRS-2 is a parent-report questionnaire of social symptoms associated with an ASD diagnosis. Symptoms are rated across several domains, including social motivation, cognition, communication, awareness, and restricted/repetitive behaviors. Domain and total scores are presented as standardized T scores. The SRS shows high sensitivities (0.74–0.80) and specificities (0.69–1.00) for ASD [53]. Analyses included SRS total T scores in order to examine the total range of ASD-related symptoms.

### Salivary testosterone collection

Salivary testosterone can be measured reliably and noninvasively utilizing small amounts of saliva, making it an ideal measure in studies of children and youth (e.g., [54, 55]). Salivary samples were collected 4 times per day from the home over 3 consecutive weekdays using established protocols [56, 57]. It has been previously demonstrated that testosterone levels are highest in the morning and decline throughout the day [54]. In children and adolescents, testosterone concentrations are significantly lower than in adults. Given the relatively young age of the participants, afternoon and evening testosterone concentrations were often below the assay’s level of detection (2.5 pg/ml), a common problem in salivary testosterone research, particularly for younger children and early adolescents [58, 59]. As such, the current study focused on morning testosterone concentrations, collected immediately upon waking and 30-min after awakening.

Samples were collected by passive drool through a straw into 2.0 ml screw cap microcentrifuge tubes distributed by Thermo Fisher Scientific. As many participants struggled to produce adequate saliva needed for assay, a salivary stimulant, namely, Trident® original flavor sugarless gum was provided. According to previous reports [58], and our own testing [60], the use of the gum does not significantly affect testosterone concentrations compared to passive drool with no stimulant. Participants were provided with direct instruction and a mini-manual of step-by-step passive drool procedures. They were instructed to avoid food and drink consumption for one hour prior to sample collection. If the participant became ill, home sampling was rescheduled until after the participant was healthy. For females, the menstrual cycle was documented, and basal testosterone was collected during the mid-Follicular to early-Luteal phase to reduce variability across the menstrual cycle [61]. Samples were refrigerated until the next study visit (approximately 3–5 days post-saliva collection), at which point the study team stored samples in an ultra-low temperature (− 80 C) freezer until being sent for assay.

### Testosterone assay

The salivary testosterone radioimmunoassay (RIA) performed in Hormone Assay and Analytical Services Core Laboratory of the Vanderbilt Diabetes Research and Training Center was developed in the laboratories of the Division of the Diabetes, Endocrinology and Metabolism, Department of Medicine, Vanderbilt University Medical Center, Nashville, TN, 37232. The primary antibody to testosterone was purchased from MP Biomedicals, Cat#: 07-189113. Testosterone-19-Carboxymethlyether-BSA was used as the antigen to generate antiserum in rabbits. The antibody is highly specific to testosterone. Cross-reactivity in the testosterone biosynthetic pathway is 5α-dihydrotestosterone (3.4%), 5α-androstane-3β,17β-diol (2.2%), 11-oxotestosteron (2.0%), 6β-hydroxytestosterone (0.95%), androstenedione (0.56%), progesterone (< 0.01%), and estradiol-17β (< 0.01%).

The assay is performed with testosterone I-125 from MP Biomedicals, Cat: #07-189121. Prior to assay, saliva was stored at – 20 °C, then thawed and centrifuged at 3460 rpm (2650 g) for 15 min to separate the aqueous component from mucins and other suspended particles. All samples were run in duplicate. The sensitivity of the assay is 0.0025 ng/ml. The inter-assay SD when a pool of human saliva was assayed repeatedly was ± 0.0013 ng/ml (*n* = 31, mean = 0.0063 ng/ml).

### Statistical analyses

For all aims, we used linear mixed-effects models to account for repeated measurements within subjects using a random intercept and considered covariates age, sex, diagnosis, period, pubertal stage, and SRS total score. For all models, unless otherwise noted, natural cubic splines with 3 degrees of freedom (4 knots) were used to allow for non-linearity in the effect of age. For hypothesis testing, we used type 2 sum of squares so that lower order terms are tested without their higher order interactions included in the model. Robust standard errors were obtained to account for model misspecification. Models were fit within R using the nlme package. We used a robust effect size index, denoted *S*, which is defined in a wide array of settings and is proportional to ½ Cohen’s *d* [62].

## Results

**Fig. 1:**
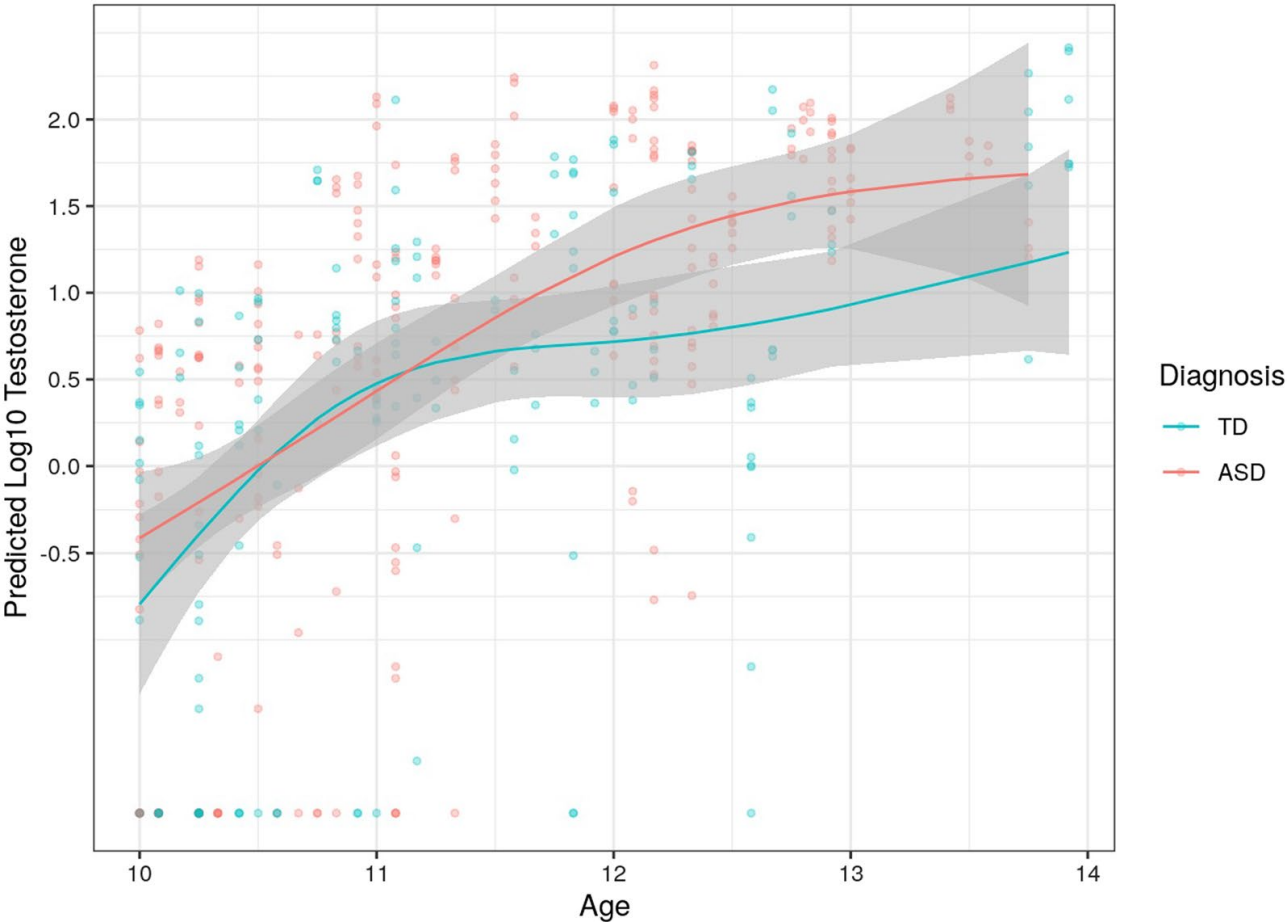
Predicted waking testosterone by age in males with and without ASD

To examine hypothesized diagnostic effects on testosterone concentrations, a linear mixed-effects model was fit with age, sex, diagnosis, period (e.g., waking vs. 30 min post-waking), and an age-by-diagnosis interaction. All these hypotheses were tested in the same model (by first testing the main effect of diagnosis and then testing the age-by-diagnosis interaction) to see whether age-related changes in testosterone differed by diagnosis. As shown in Table 2, there were significant main effects for nonlinear age (*p* < 0.001) and diagnosis (*p* = 002), where testosterone levels increased with older age and ASD diagnosis (see Additional file 1: Table S1 for parameter estimates). Next, we tested the nonlinear age-by-diagnosis interaction to investigate whether age-related changes in testosterone differed by diagnosis; this effect was not significant (*Χ*^2^ = 5.18, *df* = 3, *p* = 0.159, *S* = 0.104). While the nonlinear age-by-diagnosis was not significant, testosterone increases with age were more rapid for the ASD group (Fig. 1) and an exploratory model using linear age showed a significant age-by-diagnosis interaction with a small effect size (*Χ*^2^ = 4.72, *df* = 1, *p* = 0.030, *S* = 0.136).

**Table 2:** Type 2 sum of squares for main effects of diagnosis, sex, age, and a diagnosis*age interaction

Factor	*Χ*^2^	*df*	*p*	Effect size (*S*)
Sex	0.98	1	0.323	0.0
Age (ns)	145.59	3	**< 0.001**	0.82
Diagnosis	9.59	1	**0.002**	0.20
Period	39.61	1	**< 0.001**	0.43
Diagnosis*age (ns)	5.96	3	0.114	0.12

Significant *p* values in bold

df, degrees of freedom; ns, natural cubic splines

**Fig. 2:**
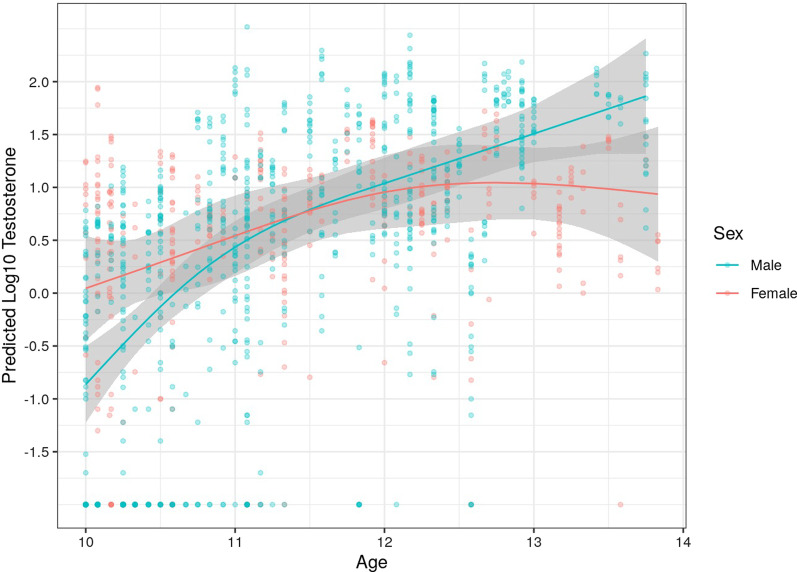
Predicted testosterone at waking for males and females

To further examine the effects of age and sex on testosterone levels, an additional interaction term between nonlinear age and sex was added to the model. This interaction term had a strong association with testosterone (*Χ*^2^ = 22.38, *df* = 3, *p* < 0.001, *S* = 0.23), but did not qualitatively change the nonlinear age-by-diagnosis interaction (*Χ*^2^ = 3.17, *df* = 3, *p* = 0.367, *S* = 0.029). To visualize the nonlinear age*sex interaction, we refit the model without the nonlinear age-by-diagnosis interaction (Table 3; Fig. 2). Lastly, a sex*diagnosis interaction term was added to examine whether sex differences in testosterone differed across ASD and TD groups; however, the interaction was not statistically significant (*Χ*^2^ = 0.02, *df* = 1, *p* = 0.879, *S* = 0.000).

**Table 3:** Type 2 sum of squares for main effects of diagnosis, sex, age, and a sex*age interaction

Factor	*Χ*^2^	*df*	*p*	Effect size (*S*)
Age (ns)	154.46	3	**< 0.001**	0.85
Sex	2.25	1	0.133	0.08
Diagnosis	3.87	1	**0.049**	0.13
Period	39.74	1	**< 0.001**	0.43
Sex*age (ns)	25.07	3	**< 0.001**	0.32

Significant *p* values in bold

df, degrees of freedom; ns, natural cubic splines

While participants were excluded if taking oral contraceptives, growth hormones, or other medications known to directly affect the HPG axis, five participants with ASD were taking Risperdal (Risperidone), which may affect steroidogenesis (see drug label at accessdata.fda.gov). To investigate whether differences in Risperidone prescription were driving sex or diagnosis differences, we refit the model removing the five participants who were taking Risperidone and the results were qualitatively identical.

The second aim of the study was to examine the effects of the pubertal stage and social functioning on testosterone levels. To investigate this aim, we added fixed effects for GB stage and SRS Total Score to the model with nonlinear age, sex, diagnosis, and nonlinear interaction terms age*sex and age*diagnosis. Complete statistical results are given in Table 4. The model showed significant main effects for GB stage (*p* < 0.001), sex (*p* = 0.042), and age (*p* < 001). The interaction term for age*sex was significant (*p* = 0.006), while there was no effect of diagnosis or age*diagnosis interaction. SRS total score did not significantly predict testosterone levels (Table 4; Additional file 1: Table S2 for parameter estimates).

**Table 4:** Type 2 sum of squares for main effects of diagnosis, sex, age, GB stage, SRS total, diagnosis*age, and sex*age

Factor	*Χ*^2^	*df*	*p*	Effect Size (*S*)
GB stage	53.12	1	**< 0.001**	0.50
SRS total	0.01	1	0.915	0.00
Age (ns)	56.39	3	**< 0.001**	0.51
Sex	4.12	1	**0.042**	0.12
Diagnosis	0.54	1	0.464	0.0
Period	38.37	1	**< 0.001**	0.42
Sex*age (ns)	12.58	3	**0.006**	0.22
Diagnosis*age (ns)	5.07	3	0.167	0.10

Significant *p* values in bold

df, degrees of freedom; ns, natural cubic splines

To determine whether results differed when including PH stage, rather than GB stage, models were repeated with PH stage as a predictor. As with GB stage, there was a significant main effect for PH stage (*Χ*^2^ = 58.29, *df* = 1, *p* < 0.001, *S* = 0.525). The interaction term between age*sex was significant (*Χ*^2^ = 13.57, *df* = 3, *p* = 0.004, *S* = 0.226), while there was no effect of diagnosis or age*diagnosis interaction (*Χ*^2^ = 2.69, *df* = 3, *p* = 0.442, *S* = 0.00. Further, SRS total score did not significantly predict testosterone levels (*Χ*^2^ = 0.319, *df* = 1, *p* = 0.572, *S* = 0.00; PH results available in Additional file 1: Tables S3 and S4).

While reported models examined testosterone levels as the outcome variable to allow for a consistent modeling framework, it is plausible that testosterone would itself predict social functioning (SRS) or pubertal development (GB/PH stage). To examine this possibility, we fit separate linear models with SRS score, GB stage, or PH stage as the outcome and testosterone as a predictor. Qualitatively, results were similar, with no significant main effect for testosterone as a predictor of SRS total and a significant effect for testosterone as a predictor of GB and PH stage. Tables of the main effects for each model can be found in Additional file 1: Tables S5, S6, and S7.

## Discussion

For decades, researchers have aimed to identify factors associated with autistic symptoms in an effort to develop and refine early diagnostic and intervention techniques. Of these efforts, the fetal androgen theory [26] proposed that exposure to increased androgen levels prenatally may increase the likelihood for later presentation of autistic-like traits [24–26]. Substantial literature has emerged to provide support for these theories—now expanded to include estrogens (prenatal steroid theory; [23])—where elevations in sex steroids, such as androstenedione and testosterone, measured via amniotic fluid sampling or in umbilical cord blood, were associated with an increase in autistic symptoms in later toddlerhood and childhood (e.g., [28, 30–32]). Furthermore, a direct study of males born between 1993 and 1999 found those with an eventual diagnosis of autism had increased steroidogenic activity, measured in the amniotic fluid, relative to typically developing controls [63]. The current study sought to extend past research by examining testosterone concentrations during a later period of high sex steroid activity, namely puberty, to determine the extent to which males and females with ASD continue to demonstrate elevated testosterone compared to TD peers.

Across several of the statistical models, a significant difference in testosterone concentrations was observed between ASD and TD youth, with elevations in the ASD group confirming our hypotheses. The results appear consistent with much of the extant literature including evidence for elevated testosterone prenatally (e.g., [63]). Furthermore, some limited findings in older children [33, 64] or adults [38] with ASD have reported elevated testosterone or testosterone precursors. In contrast, other studies have cited no significant differences, or even lower testosterone, between ASD and TD, particularly around the prepubertal or post-pubertal period [39–41]. Nevertheless, our study extends previous research in prenatal samples to support continued testosterone and HPG axis differences during the adolescent period in ASD. Critically, the dynamic nature of adolescence and pubertal development warrants careful consideration when examining hormonal differences, including testosterone.

Given the complexities of HPG axis regulation as a function of age and development, we further explored possible diagnostic and age interactions. Although testosterone levels increased with older age and appeared to do so at a more rapid rate for ASD youth (Fig. 1), there were no significant interaction effects identified between diagnostic group status and age. In other words, while the presence of ASD may influence testosterone levels, age is likely a stronger predictor of elevations in testosterone during adolescence. Indeed, it is well established that the HPG axis during puberty is activated, resulting in a rise in testosterone and other sex steroids to enable sexual maturation [14].

The most consistent finding across multiple models was the significant interaction effect between age and sex. Interestingly, females had higher testosterone at younger ages, but by age 11.5 years, males began to show higher testosterone levels compared to females, regardless of diagnosis (see Fig. 2). These findings are consistent with previous longitudinal research in TD youth ranging from 8 to 29 years demonstrating unique sex-based differences in the developmental slope for testosterone [65]. Females in the current sample demonstrated an early increase in testosterone, followed by a plateauing by mid- to-late adolescence similar to research in neurotypical populations [65, 66]. In contrast to the female trajectory described above, males showed a rapid testosterone increase which continued throughout later development stages (Fig. 2), also similar to previous reports [65]. These trajectories are consonant with known sex-based differences in hormonal expression (i.e., estrogen in females, testosterone in males [67], suggesting future research should carefully consider biological sex when examining hormonal differences between diagnostic groups.

In an effort to distinguish between adolescence (age) and pubertal effects while examining sex and diagnosis, additional models adjusted for the main effect of pubertal stage (GB and PH). As expected, more advanced pubertal stage was associated with increased testosterone. There was no main effect for diagnosis or an age-by-diagnosis interaction, suggesting ASD and TD youth did not differ in testosterone concentrations across advancing age when accounting for pubertal timing. The results were consistent when examining either GB or PH stage, showing the expected rise in testosterone levels over pubertal growth. However, there was no effect of social functioning, as measured by the SRS, on testosterone levels for any of the aforementioned models, nor did testosterone levels predict SRS scores. Nevertheless, future research will need to closely examine the complex interplay of social functioning, development, hormonal activation, and diagnostic status to enhance understanding of hormonal effects on cognition and behavior throughout the lifespan.

On the surface, these results do not appear to align with a recent study comparing prenatal testosterone (pT) to autistic traits in adolescents and young adults aged 13 to 21 [68]. Similar to our current report, there were no direct associations between pT and autistic traits. In contrast, however, exploratory analysis suggested a positive correlation between autistic traits and pT in those with earlier pubertal timing, which was stronger in males [68]. As such, there may be unique associations between pubertal timing, ASD symptoms, and biological sex. Notably, the females with ASD in the current sample evidenced advanced pubertal timing compared to TD females during early adolescence when the sample was 10 to 13 years of age [69], which may correspond with their higher levels of testosterone observed at these younger ages. Earlier pubertal timing was also observed in males with ASD albeit during middle adolescence [70]}, which again seems to align with the rise in testosterone in males. Despite this intriguing connection between pubertal testosterone, autism, and sex, there are notable differences in methods between the current study and Dooley et al. (2022), which include: sample differences (confirmed ASD diagnosis vs. self/parent-report of autistic traits), sample age (10–13 years vs. 13–21 years), testosterone effects (pubertal/activational vs. prenatal/organizational), and pubertal development (Physical Exam Tanner staging vs. PDS questionnaire), respectively. Furthermore, while the current study did not find any relationship between ASD diagnosis and testosterone at varying developmental stages, we did not explore differences according to early-, normative-, or late-pubertal timing, nor did we examine a relationship with age of menarche in females. However, future studies following the current sample across four years will be able to examine pubertal timing and tempo differences and testosterone levels throughout early-to-late adolescence. Overall, despite their differences, both studies suggest a potential overlap with regards to the association between testosterone levels, autistic traits, and pubertal timing.

The findings indicate that testosterone may play a role in the manifestation or presentation of ASD, especially during periods of dynamic hormonal changes, namely gestation and pubertal development. Inherent developmental (age, puberty) and sex-based (male, female) factors play a more prominent role in changes in testosterone levels during adolescence. While the prenatal steroid theory [23] relates to prenatal, organizational effects of testosterone in autism, the current study focused on what are likely activational, behavioral influences of androgens during pubertal development. It remains to be determined whether fetal steroid levels directly correlate with pubertal levels [24, 25] and whether testosterone may modulate the autism phenotype after fetal development [71, 72]. Future longitudinal studies of steroid levels from the prenatal to the pubertal period would provide more insight into both organizational and activational roles for testosterone in ASD. This notion is important to consider especially since findings during childhood and adolescence are mixed (e.g., [24, 31, 32, 39, 40, 68]). It is plausible there exists a more nuanced effect in which elevations in testosterone are observed in a subset of youth, such as those with earlier pubertal onset. Indeed, advanced pubertal onset has been observed in females with ASD during early adolescence [69] and more recently reported in females and males with ASD during early-to-middle adolescence [70].

To date, the vast majority of research on testosterone has focused on the impact of fetal androgens on the autism phenotype. Results suggest that diagnosis in concert with other strong developmental predictors may contribute to autistic characteristics in a subgroup of youth. Future studies with larger, well-characterized samples are warranted to explore a) the influence of biological sex and developmental factors to determine changes in testosterone levels over the pubertal transition, b) whether differences in testosterone values extend into adulthood, c) other biological explanations of testosterone differences in ASD (e.g., genetics [73], physical health/body weight), and d) what impact testosterone changes or levels may have on the ASD symptom profile throughout development.

### Limitations

There are many strengths to the study including the rigorous measurement of ASD, pubertal development, and morning salivary testosterone and a robust statistical design. Nevertheless, limitations exist. The current study sample was unequal in its male-to-female distribution, which may be notable when considering established sex-based hormonal differences, particularly during the pubertal period. Also, the sample was non-representative in terms of broader cognitive functioning and race/ethnicity; therefore, the extent to which findings generalize across the spectrum is uncertain. Finally, the detection limit in the assay prevented examining testosterone at time points other than morning samples.

## Conclusions

Overall, youth with ASD demonstrated elevated testosterone compared to TD youth. While ASD had higher testosterone than TD, there is no evidence to suggest changes in concentrations over time differ significantly beyond the expected sex-based trajectories. Similarly, age and pubertal stage were highly predictive of elevated testosterone. Finally, despite hypothesized relationships between testosterone and social functioning, particularly in ASD, it was not a significant predictor of testosterone. In summary, testosterone may play a unique role in the presentation of ASD, especially during periods of dynamic hormonal changes including puberty. Inherent developmental (age, puberty) and sex-based (male, female) factors play a more prominent role in changes in testosterone levels during adolescence. Even so, future research is warranted to determine the differential expression and impact of exposure to excess testosterone during multiple critical periods of development (e.g., prenatal, postnatal, pubertal) t for youth with ASD.

## Supplementary Information

**Additional file 1.** Excel workbook (.xlsx) for Tables S1–S7. **Table S1**: Parameter estimates of model including Sex, Nonlinear Age, Diagnosis, and Age*Diagnosis; **Table S2**: Parameter estimates of model including Diagnosis, Sex, Age, GB stage, SRS Total, Diagnosis*Age, and Sex*Age; **Table S3**: Type 2 Sum of Squares for Main Effects of Diagnosis, Sex, Age, PH stage, SRS Total, Diagnosis*Age, and Sex*Age; **Table S4**: Parameter estimates of model including Diagnosis, Sex, Age, PH stage, SRS Total, Diagnosis*Age, and Sex*Age; **Table S5**: Type 2 Sum of Squares for Main Effects of Testosterone, Diagnosis, Sex, Age, GB stage, Diagnosis*Age, and Sex*Age on SRS Total; **Table S6**: Type 2 Sum of Squares for Main Effects of Testosterone, Diagnosis, Sex, Age, Diagnosis*Age, and Sex*Age on GB Stage; **Table S7**:, Type 2 Sum of Squares for Main Effects of Testosterone, Diagnosis, Sex, Age, Diagnosis*Age, and Sex*Age on PH Stage.

## Data Availability

The datasets used and analyzed during the current study are available from the corresponding author on reasonable request.

## Abbreviations

ADOS-2: Autism Diagnostic Observation Schedule, 2nd Edition
ASD: Autism spectrum disorder
BMI: Body mass index
DHEA: Dehydroepiandrosterone
EMB: Extreme male brain
E–S: Empathizing–sympathizing
FSH: Follicle-stimulating hormone
GB: Genital (G)/breast (B)
GnRH: Gonadotropin-releasing hormone
HPA: Hypothalamic–pituitary–adrenal
HPG: Hypothalamic–pituitary gonadal
LH: Luteinizing hormone
PE: Physical examination
PH: Pubic hair
pT: Prenatal testosterone
Q-CHAT: Quantitative Checklist for Autism in Toddlers
SRS-2: Social Responsiveness Scale, 2nd Edition
TD: Typical development
WASI-2: Wechsler Abbreviated Scale of Intelligence, 2nd Edition
